# Quantum state-controlled directional spontaneous emission of photons into a nanophotonic waveguide

**DOI:** 10.1038/ncomms6713

**Published:** 2014-12-12

**Authors:** R. Mitsch, C. Sayrin, B. Albrecht, P. Schneeweiss, A. Rauschenbeutel

**Affiliations:** 1 Vienna Center for Quantum Science and Technology, Atominstitut, TU Wien, Stadionallee 2, 1020 Vienna, Austria

## Abstract

The spin of light in subwavelength-diameter waveguides can be orthogonal to the propagation direction of the photons because of the strong transverse confinement. This transverse spin changes sign when the direction of propagation is reversed. Using this effect, we demonstrate the directional spontaneous emission of photons by laser-trapped caesium atoms into an optical nanofibre and control their propagation direction by the excited state of the atomic emitters. In particular, we tune the spontaneous emission into the counter-propagating guided modes from symmetric to strongly asymmetric, where more than 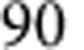
% of the optical power is launched into one or the other direction. We expect our results to have important implications for research in quantum nanophotonics and for implementations of integrated optical signal processing in the quantum regime.

## Abstract (web summary)

Nanoscale confinement in an optical fibre induces coupling between a photon’s spin and orbital angular momentum. Here, the authors use this effect to control the direction of photons spontaneously emitted from trapped caesium atoms into a nanofibre.

## Graphical abstract

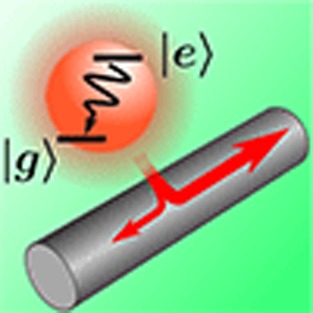

In recent years, nanophotonic devices have gained increasing importance for many applications^1^,^2^. In these structures, the light is strongly confined at the wavelength or subwavelength scale and generally exhibits a significant spin–orbit interaction (see ref. 3 and references therein). Remarkably, the local spin of the confined light can be transverse, that is, orthogonal to the propagation direction of the field^4^,^5^. Because of time reversal symmetry, this transverse spin changes sign when the direction of propagation is reversed. Recently, several experiments exploited this coupling of the local spin and the propagation direction for a directional excitation of classical electromagnetic guided model, The directional launching of plasmonic surface waves has been demonstrated^6^,^7^ and asymmetric scattering patterns of radio frequency waves have been observed in subwavelength hyperbolic metamaterials^8^. By coupling the dipole scatterers to one- or two-dimensional dielectric waveguides with subwavelength-diameter or -thickness, directional scattering of visible and near-infrared light into the waveguide modes has been achieved^9^,^10^,^11^. Lately, a similar experiment has also been realized with a near-field probe coupled to a photonic crystal waveguide^12^.

Here we take advantage of the transverse spin of strongly confined light to demonstrate the directional spontaneous emission of photons by atoms into a nanophotonic waveguide. We show that the propagation direction of these photons is controlled by the excited state of the atomic emitters. In particular, the spontaneous emission into the counter-propagating guided modes can be tuned from symmetric, as also encountered for decay in free space, to strongly asymmetric, where more than 90% of the optical power is launched into one or the other direction.

## Results

### Experimental setup

**Figure 1:**
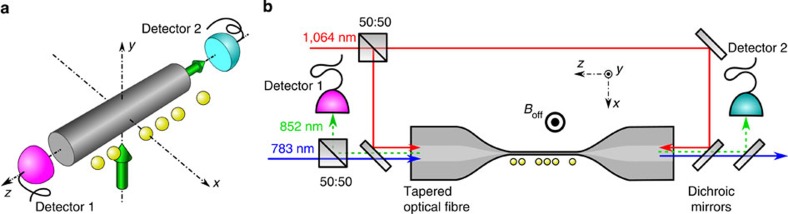
Experimental realization of a directional atom-nanofibre interface. (**a**) Schematic of the experiment: atoms (yellow spheres) are trapped on one side of the nanofibre (radius *a*) at the transverse position (*y*=0 and, here, *a*<*x*≈480 nm). A left-handed circularly polarized laser beam that propagates in the +*y*-direction (vertical green arrow) excites the atoms. The fluorescence light that the atoms emit into the nanofibre is recorded using two detectors, one at each end of the fibre. (**b**) Sketch of the setup including the tapered optical fibre (TOF), the dipole trap laser beam paths (red and blue lines), the resonant light beam paths (green dotted lines) and the atoms at the nanofibre section of the TOF. The orientation of the external homogeneous offset magnetic field *B*_off_ is indicated. 50:50 denotes a balanced polarization-independent beam splitter. The wavelengths of the light fields are indicated.

We use a small number of caesium atoms as quantum emitters. The atoms are located in the vicinity of the surface of a subwavelength-diameter silica nanofibre, see Fig. 1a. Because of this close proximity, the atoms are efficiently interfaced with the waveguide modes via their evanescent field part. Consequently, a fraction of the atomic fluorescence couples into the waveguide. The experiment is implemented using a nanofibre-based optical dipole trap for laser-cooled atoms^13^. The experimental setup is sketched in Fig. 1b. The optical nanofibre has a nominal radius of *a*=250 nm and is realized as the waist of a tapered optical fibre^14^. It enables almost lossless coupling of light fields that are guided in a standard optical fibre into and out of the nanofibre section. The nanofibre-based trap is created by sending a blue-detuned running-wave field with a free-space wavelength of 783 nm and a power of 8.5 mW and a red-detuned standing wave field at 1,064 nm wavelength with a power of 0.77 mW per beam into the nanofibre^13^. The trapping potential consists of two diametric linear arrays of individual trapping sites along the nanofibre, located 230 nm from the surface. Here only one linear array of atoms is prepared^15^. Each site contains at most one atom and provides a strong subwavelength confinement in all three dimensions^16^, considering the wavelength of 852 nm of the employed caesium transition. Thus, the local properties of the nanofibre-guided modes shape the atom—field interaction.

### Spin–orbit coupling of light in an optical nanofibre

The physical origin of the directional spontaneous emission of light into the nanofibre lies in the polarization properties of the guided modes^11^. For an atom at the position **r**, the spontaneous emission rate into one of the nanofibre modes is proportional to |d*·**u**(**r**)|^2^, where **d** is the atomic dipole operator and * denotes the complex conjugation. The coupling between the atomic emitters and a given nanofibre mode thus crucially depends on its local unit polarization vector **u** (ref. 17). For a sufficiently small fibre radius, as realized here, the optical nanofibre only guides the fundamental HE_11_ mode^17^. These strongly guided optical fields are special in the sense that they show a significant coupling of the light’s spin and orbital angular momentum^18^. The electric part of the local spin density is proportional to the ellipticity vector, which is given by the cross product *i*[u* × **u**]. In strong contrast to paraxial light fields, the local spin density is position dependent, in general not parallel to the guided field’s propagation direction, and even transverse in the case of quasi-linearly polarized guided fields^4^,^19^,^20^. Most importantly for the following, in the latter case, the local spin changes sign when reversing the propagation direction of the guided field^11^. This effect is a clear signature of the coupling of the light’s spin and orbital angular momentum. It allows us to control the direction of spontaneous emission that is coupled into the nanofibre.

**Figure 2:**
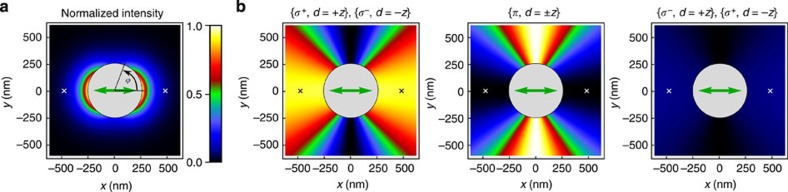
Nanofibre-guided quasi-linearly polarized basis modes. All quantities are calculated for running-wave fields with a wavelength of 852nm and a 250 nm-radius silica fibre and are plotted in the plane transverse to the nanofibre axis. The orientation of the main polarization *p*=*x* is indicated by a green double arrow. The two alternative positions of the atoms are indicated by small crosses. (**a**) Intensity distribution, normalized to the maximal intensity at the nanofibre surface. A significant fraction of the guided optical power propagates outside the nanofibre, allowing one to efficiently interface atoms with the nanofibre-guided light field. The azimuthal variation of the intensity distribution is apparent. (**b**) Polarization overlaps, 
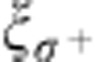
, *ξ*_*π*_ and 
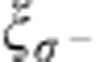
, for the specified propagation direction *d*=±*z* of the nanofibre-guided modes. The local quantization axis is chosen along **e**_*ϕ*_. The plots also apply to the *p*=*y* modes when rotated by *π*/2.

In the following, we consider the quasi-linearly polarized HE_11_ modes^17^. Four such modes, which have their main polarization *p* oriented along the *x* axis or along the *y* axis (*p*=*x* or *y*) and which propagate in the forward or backward direction (*d*=+*z* or −*z*) form a basis. The intensity profile of the quasi-linearly polarized basis modes is shown in Fig. 2a. Figure 2b shows a decomposition of the basis modes into the *σ*^+^, *π* and *σ*^−^ polarization components^17^. To account for the cylindrical symmetry of the nanofibre, we take the position-dependent direction of **e**_*ϕ*_ as the local quantization axis, where **e**_*ϕ*_ is the azimuthal unit vector. We introduce the overlaps 
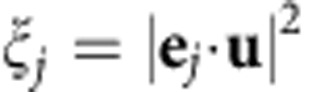
, *j*ε{*σ*^+^, *π*, *σ*^−^}, of the polarization vector ***u*** with the orthonormal basis vectors **e**_*π*_=**e**_*ϕ*_, 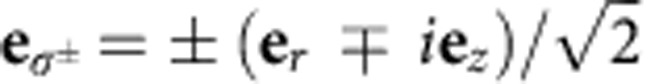. With these definitions, a non-vanishing overlap 
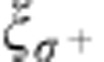
 or 
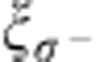
 is a signature of a transverse spin of the nanofibre-guided mode. We plot the overlaps *ξ*_*j*_ in Fig. 2b. They are constant along the nanofibre and vary only slowly in the radial direction. However, they strongly vary as a function of the azimuthal position around the nanofibre. Thus, the local spin density depends on both, the azimuthal position in the nanofibre transverse plane and the propagation direction of the mode.

For the *p*=*x* modes, at *ϕ*=0 or *π*, that is, on the right or the left side of the nanofibre in Fig. 2, respectively, 
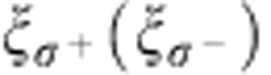
 is maximal when the propagation direction of the mode is +*z* (−*z*), see the left panel of Fig. 2b. At a distance of 230 nm from the nanofibre surface, namely at *r*=480 nm and *ϕ*=0 or *π*, we have 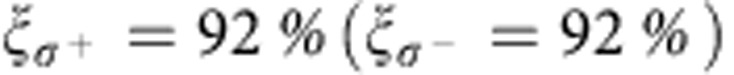. Thus, the so-called quasi-linearly polarized modes are locally almost perfectly circularly polarized, corresponding to a significant local spin density. Remarkably, this local spin is parallel to **e**_*ϕ*_, that is, it is orthogonal to the propagation direction of the mode, and points in opposite directions on opposite sides of the nanofibre. In contrast to the overlaps 
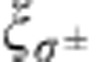
, *ξ*_*π*_ does not show a dependence on the propagation direction. For the *p*=*x* modes, *ξ*_*π*_=1 for *ϕ*=*π*/2 or 3*π*/2 and *ξ*_*π*_=0 for *ϕ*=0 or *π*. At the position *r*=480 nm and *ϕ*=0 or *π*, 
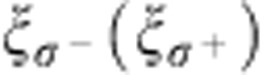
 is only 8%, when the propagation direction of the mode is +*z* (−*z*). The local polarization of the *p*=*y* modes at position (*r*, *ϕ*) corresponds to that of the *p*=*x* modes at position (*r*, *ϕ*−*π*/2). Summarizing, for *ϕ*=0 or *π*, the *p*=*y* modes are purely *π*-polarized, while the *p*=*x* modes are almost perfectly circularly polarized. The sign of the circularity is opposite for opposite propagation directions. As a consequence, a *π*-polarized photon emitted by an atom couples exclusively and equally to the two counter-propagating *p*=*y* modes, while a *σ*^±^-polarized photon preferentially couples to the *p*=*x* mode that propagates in the ±*z*-direction.

### Experimental investigation

**Figure 3:**
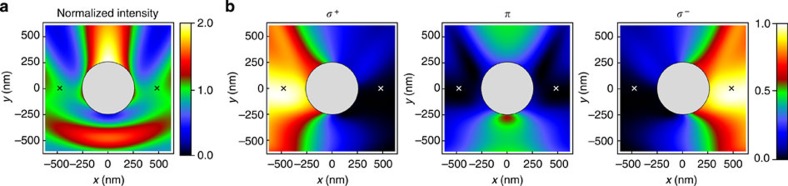
Properties of the excitation laser field. (**a**) Intensity distribution and (**b**) polarization overlaps, 
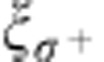
, *ξ*_*π*_ and 
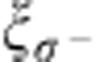
, of the excitation laser field in the vicinity of the nanofibre. The incoming field is modelled as a left-handed circularly polarized free-space plane wave that propagates in the +*y*-direction. The intensity in **a** is normalized to the incident intensity. The fibre radius is *a*=250 nm, the wavelength is *λ*=852 nm, and the local quantization axis is chosen along ***e***_*ϕ*_. The two alternative positions of the atoms are indicated by small crosses. From **b**, it is apparent that the excitation laser field is almost perfectly *σ*^−^- and *σ*^+^-polarized at the atomic positions, (*r*≈480 nm, *ϕ*=0) and (*r*≈480 nm, *ϕ*=*π*), respectively.

To experimentally study the spontaneous emission of atoms into the optical nanofibre, we prepare a single linear atomic array at the position (*r*≈480 nm, *ϕ*=0) or, alternatively, at the position (*r*≈480 nm, *ϕ*=*π*) and excite the atoms to different Zeeman states of the 6*P*_3/2_, *F*′=5 manifold: After loading atoms on both sides of the nanofibre into the trap, we optically pump them into the *m*_*F*_=0 Zeeman substate of the *F*=4 hyperfine ground state. We transfer the population in |*F*=4, *m*_*F*_=0› to |*F*=3, *m*_*F*_=0› by means of a resonant microwave *π*-pulse and remove the spurious remaining population in |*F*=4› with a push-out laser beam^21^. We then side selectively transfer the atoms back to the state |*F*=4, *m*_*F*_=0› with another resonant microwave *π*-pulse while applying a fictitious magnetic field with a strong azimuthal gradient^15^. From |*F*=4, *m*_*F*_=0›, we excite one of the four states |*F*′=5, *m*_*F*′_=±1› or |*F*′=5, *m*_*F*′_=±5›. To excite the states |*F*′=5, *m*_*F*′_=−1› and |*F*′=5, *m*_*F*′_=+1›, we prepare the atomic array at *ϕ*=0 or *π*, respectively, and shine in a resonant left-handed circularly polarized laser beam that propagates along the +*y*-direction, see Fig. 1a. With our choice of quantization axis, the latter drives *σ*^−^- or *σ*^+^-transitions for *ϕ*=0 or *π*, respectively, see Fig. 3. At the position of the atoms, the polarization of the excitation laser is almost not modified by the nanofibre. To excite the states |*F*′=5, *m*_*F*′_=−5› and |*F*′=5, *m*_*F*′_=+5›, the same optical excitation is done after an additional optical pumping stage that transfers the atoms from |*F*=4, *m*_*F*_=0› to |*F*=4, *m*_*F*_=−4› and |*F*=4, *m*_*F*_=+4›, respectively^15^. To avoid spin flips^21^ and to spectrally separate neighbouring optical transitions, a magnetic offset field of 28 G is applied in the +*y*-direction.

**Figure 4:**
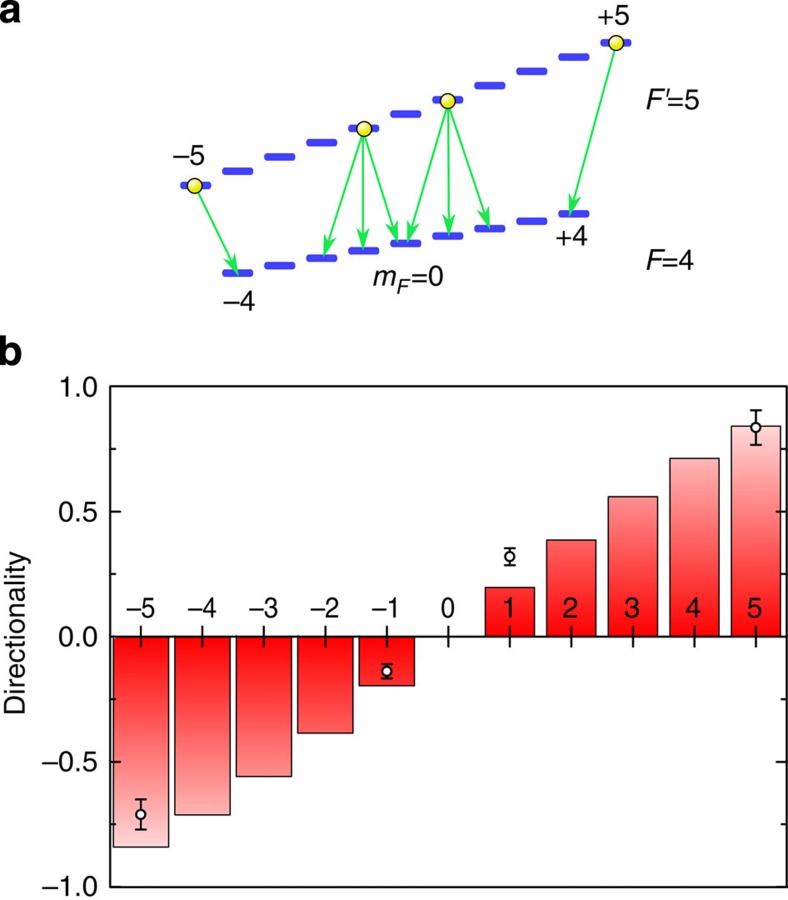
Quantum state-controlled directional spontaneous emission into an optical nanofibre. (**a**) Atomic level scheme indicating the initially excited atomic states (yellow spheres) and the decay channels (green arrows). (**b**) Directionality of the spontaneous emission into the nanofibre in dependence of the magnetic quantum number, *m*_*F*′_, of the excited 6*P*_3/2_, *F*′=5 states. Open circles: measurement results averaged over 3,200 and 4,000 experimental runs for *m*_*F*′_=±5 and *m*_*F*′_=±1, respectively. The error bars correspond to s.e. deviation estimation. Bars: theory prediction (see text). The experimental data and theory are in good agreement.

The involved atomic levels and transitions are shown in Fig. 4a. We first consider the outermost Zeeman states |*F*′=5, *m*_*F*′_=−5› and |*F*′=5, *m*_*F*′_=+5›. On decay from these states, the atoms only emit *σ*^−^- and *σ*^+^-polarized light, respectively, which exclusively couples to the *p*=*x* modes. The *σ*^−^- and *σ*^+^-polarized photons should then predominantly be emitted into the −*z*- and +*z*-directions, respectively. Two single photon counting modules, called detector 1 and 2 in the following, one at each end of the tapered optical fibre, are used to record the number of photons that are coupled into the nanofibre, see Fig. 1. The measurement interval is limited by the drop of the signal after about 10 μs, which we attribute to the loss of atoms from the trap due to photon recoil heating. We sum up all recorded photon counts individually for each detector and correct for the optical losses of the setup. In Fig. 4b, we plot the measured directionality, defined as *D*=*η*_1_−*η*_2_, as a function of *m*_*F*′_. Here *η*_1_ and *η*_2_ are the fractions of the total incoupled nanofibre-guided light that are detected by detector 1 and 2, respectively. For decay from the state |*F*′=5, *m*_*F*′_=+5›, detector 1 (receiving light that propagates in the +*z*-direction) records a significantly larger number of photons (*η*_1_=92(3)%) than detector 2, resulting in a directionality of *D*=0.84(7). The main propagation direction of the incoupled light is reversed for decay from the state |*F*′=5, *m*_*F*′_=−5›. In this case, detector 2 records the larger number of photons (*η*_2_=86(3)%), resulting in a directionality of *D*=−0.71(6).

We now excite the atoms to the |*F*′=5, *m*_*F*′_=+1› or the |*F*′=5, *m*_*F*′_=−1› state, which can spontaneously decay via a *σ*^−^, *π* or *σ*^+^ transition, leading to the emission of a photon with the corresponding polarization. Compared with the decay from the outermost states, the emission into the nanofibre is now almost balanced. We find *D*=0.32(3) and *D*=−0.14(3) for *m*_*F*′_=+1 and *m*_*F*′_=−1, respectively. These smaller values are theoretically expected: the probabilities for the emission of *σ*^−^, *π* and *σ*^+^ light for a decay from the |*F*′=5, *m*_*F*′_=±1› states are 
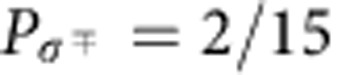
, *P*_*π*_=8/15 and 
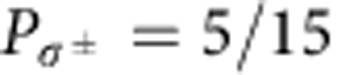
. As already discussed, the emitted *π*-polarized photons couple symmetrically into the waveguide. They thus yield the same signal on the two detectors and reduce the directionality of any emission into the nanofibre. Moreover, as *σ*^−^- and *σ*^+^- polarized photons are emitted with similar probabilities, the emission rates into the counter-propagating modes of the nanofibre are almost equal.

### Comparison with theory

Figure 4b shows the theoretically expected directionality for all *m*_*F*′_ states of the 6*P*_3/2_, *F*′=5 manifold^17^,^20^ as coloured bars. The model assumes the absence of collective scattering, which is well justified in the present situation^20^. It also takes into account the fact that the intensities of the *p*=*x* and *p*=*y* modes are not equal at the position of the atoms^20^, see Fig. 2a. Our prediction is in good agreement with the experimental results where the residual deviation can be explained by imperfect optical pumping and a slight misalignment of the excitation laser beam. For *m*_*F*′_=+5, we reach the theoretical maximum of directionality for |*m*_*F*′_›-states of *D*=0.84. Note, however, that unit directionality can in principle be reached by preparing the atom in a suitable quantum superposition of |*m*_*F*′_›-states for which the emission amplitudes into a given propagation direction cancel^11^.

## Discussion

Summarizing, we demonstrate that state preparation in conjunction with transverse spin of light allows one to control the symmetry of the spontaneous emission of quantum emitters into a nanophotonic waveguide. Our work reveals a powerful new means of tailoring light—matter interaction in the quantum regime. For example, it provides a direct way of creating entanglement between the internal state of an atom after decay and the path of a single fibre-guided photon. The presented effects are universal in the sense that they should also occur for other strongly confined optical fields^2^,^22^ and for other quantum emitters, for example, integrated photonic waveguides^1^ coupled to quantum dots^23^,^24^. We therefore expect our findings to have an important impact on integrated optical signal processing in the quantum regime. Moreover, when considering absorption instead of spontaneous emission, their complex internal level structure makes atoms unique near-field probes. This should allow one to map out, for example, via optical pumping, the extraordinary spin properties of confined light without altering the local mode structure^15^. Finally, our work represents an important step towards the realization of a cascaded quantum-optical network^25^. In such networks, the non-equilibrium dynamics of emitters that are dissipatively coupled to the chiral bath of guided photons should generate complex entangled states like chains of quantum spin dimers^26^.

## Author contributions

R.M. and C.S. equally contributed to this work. R.M., C.S. and B.A. performed the experiment. R.M., C.S and P.S. analysed the data. R.M., C.S., P.S. and A.R. wrote the manuscript. All the authors discussed the results and reviewed the manuscript.

## Additional information

**How to cite this article**: Mitsch, R. *et al*. Quantum state-controlled directional spontaneous emission of photons into a nanophotonic waveguide. *Nat. Commun.* 5:5713 doi: 10.1038/ncomms6713 (2014).
